# Protective Metabolic Effects of Chickpea Sprout Against Obesity-Induced Insulin Resistance and Hypoestrogenism in Rats

**DOI:** 10.3390/molecules30244673

**Published:** 2025-12-05

**Authors:** Patrick Mailloux-Salinas, Cristian Jiménez-Martínez, David Julian Arias-Chávez, Arturo Armando Gordillo-Bernal, María Stephanie Cid-Gallegos, Liliana Alamilla-Beltrán, Guadalupe Bravo

**Affiliations:** 1 Departamento de Ingeiería Bioquimica, Escuela Nacional de Ciencias Biológicas, Instituto Politécnico Nacional, Av. Wilfrido Massieu 399, Nueva Industrial VallejoGustavo A. Madero, Mexico City 07700, Mexico; patrickms@live.com.mx (P.M.-S.); cid.gallegos@gmail.com (M.S.C.-G.); lalamilla@ipn.mx (L.A.-B.); 2 Department of Pharmacobiology, Center for Research and Advanced Studies of the National Polytechnic Institute, South Campus, Calz. de los Tenorios 235, Coapa, Granjas Coapa, Tlalpan, Mexico City 14330, Mexico; mershall_andy@hotmail.com; 3 Pharmacology and Food Therapeutics, Dr. José Quintín Olascoaga Moncada School of Dietetics and Nutrition ISSSTE, Callejón vía San Fernando 1, San Pedro Apóstol, Tlalpan, Mexico City 14070, Mexico; djariasch@gmail.com

**Keywords:** obesity, hypoestrogenism, insulin resistance, chickpea sprouts, isoflavones, biochanin A

## Abstract

Obesity and menopausal hypoestrogenism interact in a way that worsens insulin resistance and increases the risk of metabolic diseases. This study evaluated the effects of a diet composed of liquid chickpea sprouts (CS) on these problems. Sixty-four female Wistar rats were assigned to four experimental groups: a control group (Ctrl); a hypoestrogenic (HE) group, induced by ovariotomy; an obese (Ob) group, fed a high-sucrose diet; and a hypoestrogenic-obese (HE-Ob) group. Each group was subdivided into animals treated with chickpea sprouts (CS, 0.9 g/kg/day) or with a vehicle for four weeks. The results showed that CS significantly improved glucose tolerance and restored insulin sensitivity, normalizing the HOMA-IR index in both the Ob and HE-Ob groups. In addition, CS reduced serum triglycerides, reversed hepatic steatosis, and caused a favorable redistribution of adipose tissue, leading to decreased mesenteric fat accumulation. In conclusion, chickpea sprouts have protective metabolic effects by improving glucose homeostasis, reducing blood lipids, and mitigating liver damage in an estrogen-deficient model of obesity. These findings support the potential of chickpea sprouts as a dietary intervention to help prevent metabolic complications in obese postmenopausal women.

## 1. Introduction

Obesity presents a paramount challenge to global public health, with its prevalence having tripled since 1975. Projections indicate that by 2030, over half of the world’s population will be overweight or obese [1]. This sustained increase fuels an alarming rise in metabolic disorders, including insulin resistance, type 2 diabetes, and metabolically dysfunctional adipose-associated liver disease (MASLD), which shares a common pathophysiological anchor with adipose tissue dysfunction. [2]. Far from being a passive fat reservoir, adipose tissue is now recognized as an endocrine organ that modulates energy homeostasis and insulin sensitivity through adipokines like leptin, adiponectin, and resistin [3]. In obesity, however, hypertrophic adipocyte expansion induces a state of hypoxia, oxidative stress, and chronic low-grade inflammation, which disrupts insulin signaling [4]. This proinflammatory cascade, driven by cytokines such as IL-6, TNF-α, and MCP-1, promotes macrophage infiltration and activates the NF-κB/JNK pathway, ultimately contributing to systemic insulin resistance [5].

On the other hand, hypoestrogenism—resulting from aging, menopause, or surgery—exacerbates metabolic dysfunction. Estrogens play a critical role in regulating adipose tissue distribution, fatty acid oxidation, and mitochondrial function [6]. A deficiency in these hormones triggers a central redistribution of fat, leading to increased visceral adiposity, dyslipidemia, insulin resistance, and hepatic disorders [7]. This metabolic decline is partly driven by the loss of estrogen signaling through estrogen receptor alpha (Erα) receptors in the liver and skeletal muscle, which directly impairs glucose uptake and insulin sensitivity, thereby promoting postmenopausal metabolic syndrome [8].

Obesity and hypoestrogenism interact to generate metabolically deleterious synergy, driving tissue dysfunction in muscle, liver, and adipose tissue. This combined effect accelerates lipogenesis, insulin resistance, and the progression to MASLD [8]. As shown in experimental models, estrogen deficiency worsens liver inflammation and fat accumulation, while excess free fatty acids impair insulin signaling, thereby creating a vicious metabolic cycle [6].

Current pharmacological treatments, despite their advances, fail to address this complex pathology fully. Agents like metformin, pioglitazone, GLP-1 receptor agonists, and SGLT2 inhibitors, while effective for glycemic control, are associated with clinical limitations that affect their safety and long-term adherence [9,10,11,12,13]. Consequently, there is a clear demand for novel or adjunctive strategies that enhance insulin sensitivity while safeguarding metabolic, cardiovascular, and hepatic health.

Given these limitations, the search for alternative therapeutic strategies has intensified, with particular interest in natural compounds that possess antioxidant, anti-inflammatory, and insulin-sensitizing properties. Among these, legume-derived isoflavones have demonstrated beneficial metabolic effects, including improved insulin sensitivity, and reduced hepatic steatosis [14]. A prominent isoflavone, biochanin A (BCA), has shown significant antidiabetic potential in experimental models. Its mechanisms of action include the activation of SIRT1/AMPK, PI3K/Akt, and PPAR-α/γ pathways to improve glycolipid homeostasis, alongside reducing oxidative stress and systemic inflammation via modulation of NF-κB and Nrf2/HO-1 signaling [15,16,17,18,19].

However, these findings are predominantly based on studies using purified BCA, overlooking the dietary context and potential interactions with other bioactive compounds present in whole foods. In contrast, chickpea sprouts represent a complex nutritional matrix, rich in phytoestrogens, saponins, phenolic compounds, and soluble fiber, which may synergistically modulate the metabolic effects of BCA. Germination dramatically enhances this potential; for instance, chickpea sprouting has been shown to increase isoflavone content, including BCA, by over 100-fold compared to ungerminated seeds [20]. One further study demonstrated that under optimal conditions, BCA levels in 8-day-old sprouts can be approximately 130 times higher than in the raw seed [21]. This evidence suggests that consuming chickpea sprouts may constitute a multifactorial nutritional intervention, where the combined action of its bioactive compounds could yield synergistic benefits that surpass those of an isolated molecule.

The present study aims to evaluate the effect of a liquid diet consisting of chickpea sprouts on regulating the functional axis between tissue sensitivity to insulin (hepatic and adipose) and the pancreatic secretory response, with the goal of determining its potential in preventing metabolic and hepatic alterations associated with obesity and hypoestrogenism.

## 2. Results

### 2.1. Development of the Obesity Model

**Figure 1:**
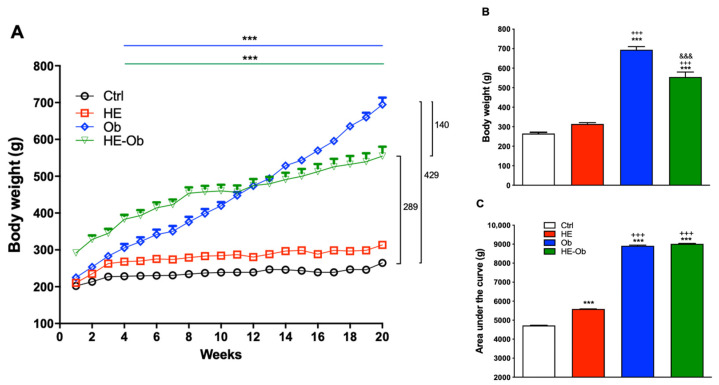
(**A**) Time course of weight gain (g) of the different experimental conditions during 20 weeks of obesity induction. (**B**) Weight of the animals under each of the experimental conditions at the end of the induction. (**C**) Area under the curve of the time courses of each of the conditions. Data are presented as the mean ± SE *n* = 8. Ctrl group vs. *** = *p* < 0.001, HE group vs. +++ = *p* < 0.001, Ob-Veh group vs. &&& = *p* < 0.001. Post hoc Tukey.

As shown in Figure 1, animals fed the hypercaloric diet (Ob and HE-Ob groups) exhibited greater weight gain during the first 4 weeks compared to the Ctrl group. Notably, the HE-Ob group showed greater weight gain during the first 8 weeks; however, this trend was attenuated after week 10 (Figure 1A). After 20 weeks of induction, the Ob group recorded the highest final weight gain, with significant differences not only compared to the Ctrl group, but also to the HE and HE-Ob groups (Figure 1B). These results are corroborated in the analysis of the area under the curve of the different experimental conditions (Figure 1C).

### 2.2. Glucose Tolerance Curves (Acute Treatment)

**Figure 2:**
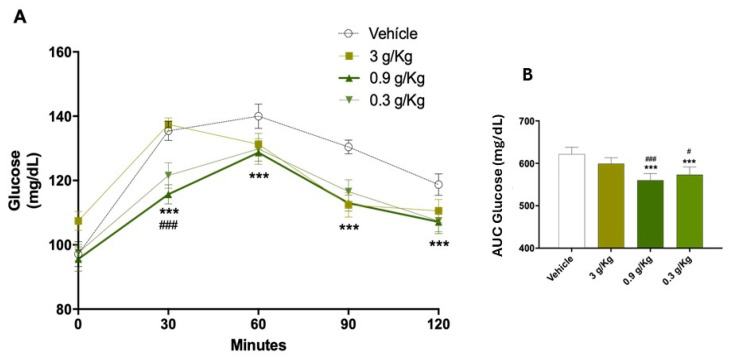
(**A**) Glucose tolerance curve of the different doses (3, 0.9, 0.3 g/kg) administered with the nine-day liquid meal diet of chickpea sprouts in normal weight animals. (**B**) Area under the curve (AUC). The data are presented as the mean ± S.E., *n* = 8. Ctrl vs. *** *p* < 0.001, 3 g/kg vs. # *p* < 0.05, ### *p* < 0.001. Post hoc Tukey.

After establishing the obesity model, the hypoglycemic effect of acute administration of three doses of chickpea sprouts (CS) was evaluated. Based on the report by Li et al. [22], who observed an effect at 3 g/kg of chickpea extract in a type 2 diabetes model, two additional lower doses (3, 0.9, and 0.3 g/kg) were tested. The results showed that the 0.9 g/kg dose induced the most significant reduction in glycemia (Figure 2A,B). This optimal effect could be attributed to a synergistic interaction among the various bioactive compounds produced during germination. Based on this finding, the dose of 0.9 g/kg was selected for the subsequent subchronic administration phase in the four experimental groups: Ctrl, HE, Ob, and HE-Ob.

### 2.3. Effect of Treatment on Body Weight and Caloric Intake

The analysis of metabolic parameters (Table 1) revealed significant differences between the experimental groups, influenced by both physiological condition and treatment with chickpea sprouts (CS). The hypercaloric diet groups (Ob-Veh and HE-Ob-Veh) showed significantly higher body weight (*p* < 0.05) than the Ctrl-Veh and HE-Veh groups, confirming the efficacy of the obesity induction model. However, the administration of CS did not produce significant changes in this parameter when comparing subgroups administered with vehicles within the same physiological condition.

In terms of food intake, the Ob-Veh and HE-Ob-Veh groups showed a reduction of around 50% compared to the Ctrl-Veh group, a phenomenon consistent with the high energy intake of their diet. Chickpea sprouts (CS) treatment significantly reduced food intake, but this effect was exclusive to the hypoestrogenic group (HE-CS). Fluid intake, in contrast, remained unchanged across all experimental groups.

In terms of raw intake, the Ob-Veh and HE-Ob-Veh groups had a significantly higher total caloric intake than the groups with a balanced diet (Ctrl-Veh and HE-Veh), reflecting the high energy density of their diet. Treatment with CS reduced total kcal intake in the balanced diet groups (Ctrl-CS and HE-CS) but did not change this parameter in the hypercaloric diet groups (Ob-CS and HE-Ob-CS). Macronutrient analysis showed that in the Ctrl-CS and HE-CS groups, treatment significantly decreased absolute protein and fat intake. In contrast, the Ob-CS and HE-Ob-CS groups had markedly higher carbohydrate intake. Finally, the percentage distribution of energy showed a dramatic contrast between the diets: the balanced diet groups maintained a constant distribution of macronutrients that CS did not alter, while the hypercaloric diet groups showed a predominance of carbohydrates (up to 87% of total energy intake) that was also unchanged by the treatment with chickpea sprouts.

### 2.4. Effect of the Hypercaloric Diet and the Treatment of Chickpea Sprouts (CS) for Nine Days on the Difference in Relative Weight of the Organs

Table 2 shows the relative organ weights for the different experimental groups. As shown, analyses of the relative weights of the organs revealed specific alterations induced by the other experimental conditions. Both the HE-Veh and HE-Ob-Veh groups exhibited a significant decrease (*p* < 0.05) in relative liver weight compared to the Ctrl-Veh group, an effect that was not modified by CS treatment. In the kidney, the obese groups (Ob-Veh and HE-Ob-Veh) showed a significant reduction, more pronounced in the HE-Ob-Veh group, suggesting a combined effect of obesity and estrogen deficiency. Treatment with CS did not alter this parameter. The weight of the heart remained unchanged in all conditions. In contrast, treatment with CS increased the relative pancreas weight in the Ctrl-CS and HE-CS groups, an effect absents in the groups with a hypercaloric diet (Ob-CS and HE-Ob-CS). Marked soleus muscle atrophy was observed exclusively in the HE-Ob-Veh group, which was not reversed by CS (HE-OB-CS) treatment. In terms of adipose tissue, obesity was associated with a significant increase in subcutaneous and perirenal fat. Treatment with CS altered subcutaneous accumulation only in the Ob-CS group compared to its vehicle (Ob-Veh), in addition to enhancing perirenal accumulation in these same animals (Ob-CS). Notably, the HE-Ob-Veh group accumulated significantly more perirenal adipose tissue than the Ob-Veh group. This distinct fat distribution pattern may indicate a different metabolic adaptation, potentially associated with the comparatively less severe insulin resistance observed in this group (as shown by the HOMA-IR index). However, the treatment did not affect this condition (HE-Ob-CS) in this parameter, as observed in (Ob-CS vs. Ob-Veh). In addition, the fat accumulated in the mesentery was higher in the Ob-Veh and HE-Ob-Veh rats, indicating that the administration of the CS treatment in these conditions (Ob-CS and HE-Ob-CS) no longer presented differences compared to the Ctrl-Veh and HE-Veh rats, resulting in a decrease in its accumulation in this area.

### 2.5. Hypoglycemic and Lipid-Lowering Effect

**Figure 3:**
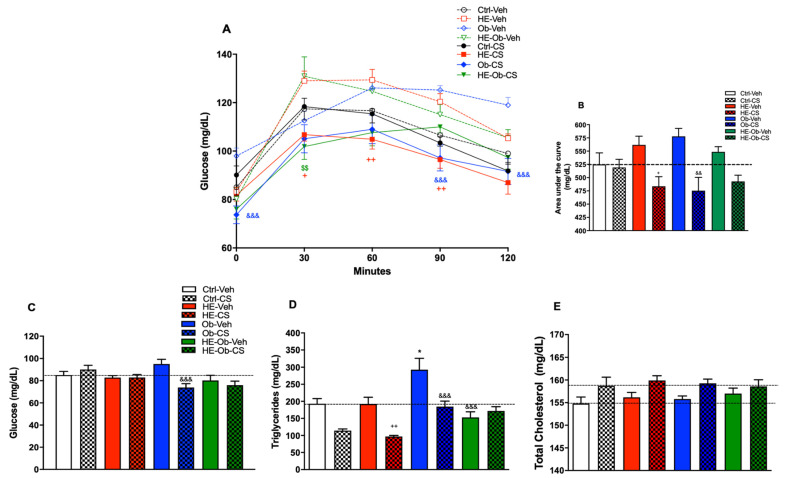
(**A**) Glucose tolerance curve, (**B**) Area Under the Curve, (**C**) Glucose, (**D**) Triglycerides and (**E**) Cholesterol (0.9 g/kg) was administered at the end of a four-week treatment, combined with a nine-day chickpea sprout (CS) liquid-flour diet. Data are presented as the mean (*n* = 8) ± S.E. Ctrl-Veh vs. * = *p* < 0.05, HE-Veh vs. + = *p* < 0.05, ++ = *p* < 0.01, Ob-Veh vs. && = *p* < 0.01, &&& = *p* < 0.001, HE-Ob-Veh vs. $$ = *p* < 0.01. Post hoc Tukey.

At the end of the subchronic CS administration regimen, which lasted four weeks, the metabolic response was assessed by OGTT. The results showed that the treatment did not induce significant alterations in the Ctrl-CS group. In contrast, statistically significant differences were identified in the HE-CS, Ob-CS, and HE-Ob-CS groups at various points on the glucose curve compared to their respective vehicles. This finding was corroborated by the area under the curve (AUC) analysis, which revealed a hypoglycemic effect of CS in the HE-CS and Ob-CS groups. However, in the HE-Ob-CS group, only a trend towards reduced glycemia was observed, which did not reach statistical significance (Figure 3).

Regarding baseline biochemical parameters, no significant changes in glucose and cholesterol concentrations were detected in most experimental groups. The exception was the Ob-CS group, in which treatment resulted in a significant decrease in fasting blood glucose levels. On the other hand, triglyceride levels were found to be elevated in the Ob-Veh group compared to the Ctrl-Veh group, reflecting diet-induced obesity-associated dyslipidemia. It should be noted that CS exerted a significant reducing effect on this parameter in the HE-CS and Ob-CS groups, suggesting a protective role of treatment against obesity-induced hypertriglyceridemia.

### 2.6. Restoring Glucose Metabolism

**Figure 4:**
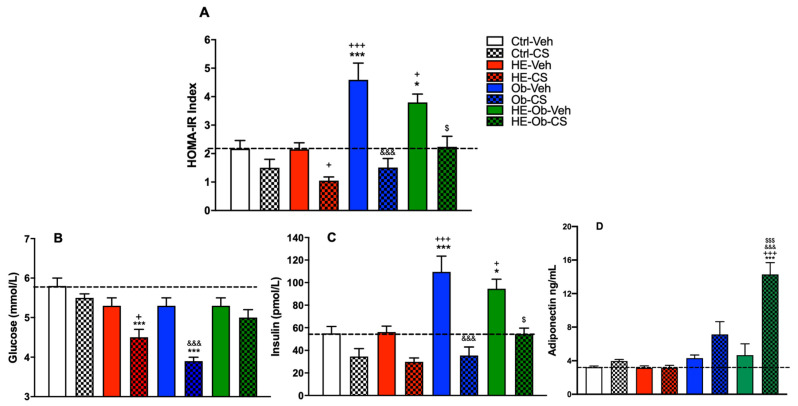
Determinations of (**A**) HOMA-IR index, (**B**) Glucose, (**C**) Insulin, and (**D**) Serum adiponectin at the end of treatment (0.9 g/Kg) for four weeks, administered with nine-day chickpea sprout flour (CS). Data are presented as the mean (*n* = 8) ± S.E. Ctrl-Veh group vs. * = *p* < 0.05, *** = *p* < 0.001, HE-Veh group vs. + = *p* < 0.05, +++ = *p* < 0.001, Ob-Veh vs. &&& = *p* < 0.001, HE-Ob-Veh vs. $ = *p* < 0.05, $$$ = *p* < 0.001. Post hoc Tukey.

Figure 4 shows the values of the HOMA index (model for the evaluation of homeostasis (Figure 4A), glucose (Figure 4B), insulin (Figure 4C), and serum adiponectin (Figure 4D) in the different experimental groups. The Ctrl-Veh and HE-Veh groups showed similar baseline values of HOMA, glucose, and insulin. However, administering chickpea sprouts to hypoestrogenic animals (HE-CS) resulted in a notable reduction in the HOMA index, mainly due to a trend toward lower insulin and glucose levels.

The Ob-Veh group had the most altered metabolic profile, characterized by a marked increase in the HOMA index and insulin levels compared to the Ctrl-Veh group, despite maintaining normal glucose levels. The treatment intervention (Ob-CS) significantly reversed this condition, normalizing both the HOMA index and insulin levels, and reducing glucose levels below those of the Ctrl-Veh group. This group also showed a tendency to increase adiponectin levels. The group with the combined condition of hypoestrogenism and obesity (HE-Ob-Veh) showed less severe insulin resistance than the Ob-Veh group, but it was still significant. Treatment in HE-Ob-CS resulted in partial improvement, reducing the HOMA index and insulin levels to levels like those of the Ctrl-Veh group, without a drastic reduction in glucose. This group stood out for the most pronounced increase in adiponectin levels, with a value much higher than those of the other groups.

These results demonstrate the powerful beneficial effects of CS on metabolic parameters associated with insulin resistance. First, it is observed that CS consistently exerts an insulin-sensitizing effect across all experimental models. The more pronounced reduction in the HOMA index in the Ob-CS and HE-Ob-CS groups suggests that the treatment is particularly effective in challenging metabolic conditions, such as obesity and hypoestrogenism. The normalization of insulin levels in these groups, even below control values, indicates a significant improvement in peripheral insulin sensitivity.

The mechanism behind this effect appears to be linked, at least in part, to the modulation of adiponectin. This adipokine, known for its insulin-sensitizing and anti-inflammatory properties, experienced a notable increase in the Ob-CS groups and, extraordinarily, in the HE-Ob-CS group. The correlation between CS administration, increased adiponectin levels, and improved metabolic profile strongly suggests that CS acts by enhancing adipose tissue function. It is interesting to note that in the HE-Ob-CS group, the improvement in insulin sensitivity was not accompanied by marked hypoglycemia, unlike in the Ob-CS group. This suggests that the CS may achieve a more stable metabolic balance under complex pathophysiological conditions, possibly through multiple pathways.

### 2.7. Morphological and Histological Evaluation

**Figure 5:**
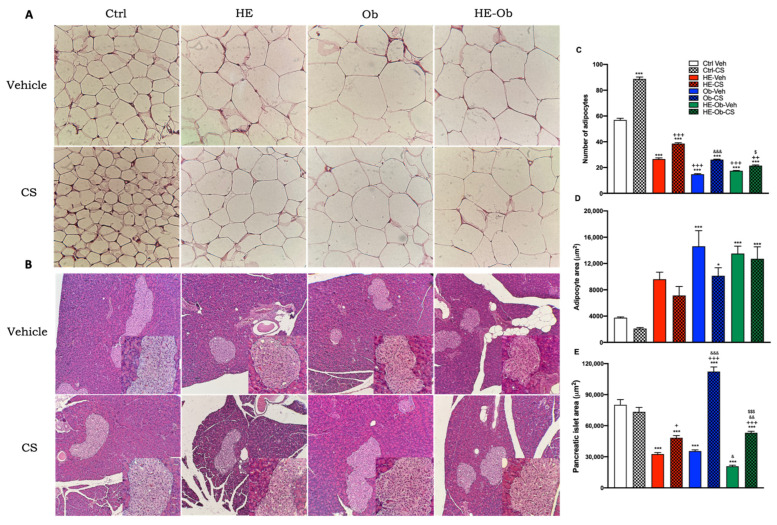
Effects of treatment on the histology and morphometry of visceral (perirenal) and pancreatic adipose tissue. (**A**) Hematoxylin-eosin histological staining of retroperitoneal adipose tissue with 10 and 40× magnifications, 5 μm microtome sections. (**B**) Hematoxylin-eosin histological staining of pancreatic tissue with magnifications of 10 and 40×, microtome slices of 4 μm. (**C**) The number of adipocytes observed per field in five zones. (**D**) area of five cells per field of the different regions analyzed. (**E**) area of five islets per field of the different areas analyzed. Data are presented as the mean (*n* = 8) ± S.E. Control vs. * = *p* < 0.05, *** = *p* < 0.001, hypoestrogenic (HE) vs. + = *p* < 0.05, ++ = *p* < 0.01, +++ = *p* < 0.001, Obese (Ob) vs. & = *p* < 0.05, && = *p* < 0.01, &&& = *p* < 0.001, hypoestrogenic Obese (HE-Ob) vs. $ = *p* < 0.05, $$$ = *p* < 0.001, Post hoc Tukey.

Figure 5A, B evaluate the histomorphological changes in adipose and pancreatic tissue, showing the number of adipocytes (Figure 5C), the mean area of adipocytes (Figure 5D) and the area of the pancreatic islets (Figure 5E) in the different experimental groups.

In adipose tissue, the Ctrl-Veh group had a baseline amount of moderately sized adipocytes. Chickpea shoot supplementation (Ctrl-CS) significantly increased the number of adipocytes and significantly reduced their individual area. Groups with metabolic challenges, such as the hypoestrogenic (HE-Veh) and obese (Ob-Veh) groups, showed a drastic reduction in adipocyte number and a marked increase in adipocyte size (hypertrophy). Treatment with CS in these groups (HE-CS and Ob-CS) partially reversed this trend, increasing the amount of adipocyte count and reducing adipocyte area. This effect was particularly evident in the Ob-CS group. The HE-Ob-Veh group showed severe hypertrophy, which was only mildly attenuated with treatment (HE-Ob-CS).

With respect to the pancreas, the islet area was significantly reduced in the HE-Veh, Ob-Veh, and HE-Ob-Veh groups compared to the Ctrl-Veh group. The treatment (HE-CS, Ob-CS, and HE-Ob-CS) showed a protective or restorative effect. While in the Ctrl-CS and HE-CS groups the effect was modest, in the Ob-CS group an extraordinary increase in islet area was observed, even exceeding that of the Ctrl-Veh group. The HE-Ob-CS group also showed substantial recovery of islet area compared to their untreated group (HE-Ob-Veh).

The results reveal the profound impact of CS on adipose tissue remodeling and on the preservation of endocrine pancreas morphology.

The most notable transformation in visceral adipose tissue suggests that CS promotes adipogenesis (increased adipocyte number) and inhibits adipocyte hypertrophy (reduced adipocyte area). This finding is crucial, as adipocyte hypertrophy, observed in the HE-Veh, Ob-Veh, and HE-Ob-Veh groups, is strongly associated with adipose tissue dysfunction, inflammation, and insulin resistance. By favoring a phenotype of more numerous and smaller adipocytes (hyperplasia), CS likely restores healthy metabolic function to adipose tissue. This mechanical explanation accounts for the results in Figure 4, where improvements in insulin sensitivity and increases in adiponectin correlate with the normalization of adipose morphology.

In the pancreas, the reduction in islet area in groups with metabolic alterations reflects possible stress or damage to β insulin-producing cells. The ability of treatment, especially in the Ob-CS group, to normalize and even significantly expand the islet area suggests a protective or regenerative effect on the endocrine pancreas. This compensatory expansion could be a direct response to the improvement in insulin sensitivity induced by CS, reducing the demand for hyperinsulinemia and allowing structural recovery. Partial recovery in the HE-Ob-CS group indicates that, in complex pathophysiological conditions, CS still exerts a beneficial effect. However, the hormonal environment may modulate the magnitude of the response.

**Figure 6:**
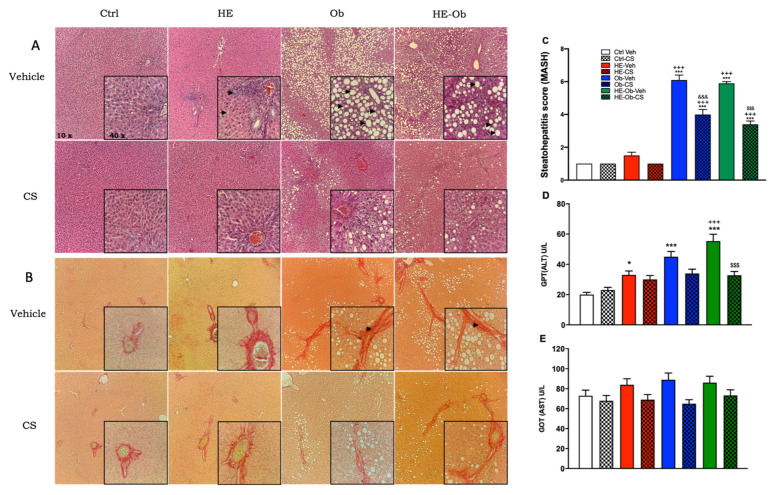
Effects of treatment on liver tissue histology and morphometry. (**A**) Hematoxylin-eosin histological stain of tissue with magnification of 10 and 40×, microtome slices of 4 μm. (**B**) Syrian red (or picrosirius red) histological staining of liver tissue with magnifications of 10× and 40×. (**C**) Score of the degree of steatohepatitis in the different areas analyzed. (**D**) Alanine aminotransferase (ALT) concentration, (**E**) Aspartate aminotransferase (GOT) concentration. Data are presented as the mean (*n* = 8) ± S.E. Ctrl vs. * = *p* < 0.05, *** = *p* < 0.001, hypoestrogenic (HE) vs. +++ = *p* < 0.001, Obese (Ob) vs. &&& = *p* < 0.001, hypoestrogenic Obese (HE-Ob) vs. $$$ = *p*< 0.001, Post hoc Tukey.

Figure 6A,B show the data from the evaluation of liver tissue, which indicate the degree of steatohepatitis associated with metabolic dysfunction (MASH, Figure 6C). The serum levels of liver enzymes ALT (GPT, Figure 6D) and AST (GOT, Figure 6E) are also presented as markers of hepatocellular damage. Histological analysis revealed that the Ctrl-Veh and Ctrl-CS groups had a healthy liver (MASH = 1). The HE-Veh group exhibited sinusoidal congestion and mild portal inflammation (MASH = 1.5), as biochemically reflected by a moderate increase in ALT and GOT. In contrast, the Ob-Veh and HE-Ob-Veh groups developed severe MASH, with scores of 6.1 and 5.9, respectively. Macro vesicular steatosis, balloon hepatocytes, sinusoidal compression, and the appearance of pericellular fibrosis histologically characterized this condition. This structural damage correlated with higher serum levels of ALT and GOT, indicating significant necroinflammation and hepatocellular damage.

Treatment with CS showed a remarkable hepatoprotective effect. In the Ob-CS and HE-Ob-CS groups, the MASH score dropped considerably to 4 and 3.4, respectively. Histologically, this corresponded to a transition from macrovesicular to microvesicular steatosis, accompanied by a reduction in damage. Biochemically, this effect was confirmed by a significant decrease in ALT and GOT levels, which brought them closer to the values of the Ctrl-Veh group. The CS also normalized the ALT enzyme in the HE-CS group.

The results in Figure 6 demonstrate the ability of chickpea sprouts (CS) to attenuate the development and severity of steatohepatitis associated with metabolic dysfunction (MASH) induced by hypoestrogenism and obesity. The correlation between histopathological findings (steatosis, inflammation, and fibrosis) and the elevation of ALT and GOT transaminases in the Ob-Veh and HE-Ob-Veh groups confirms the presence of active hepatocellular damage, characteristic of the progression from MASLD to MASH. ALT is established as a sensitive marker that faithfully reflects the degree of necroinflammation observed under the microscope.

The therapeutic effect of CS manifests itself on two levels. First, at the histological level, it induces structural improvement by reducing the degree of steatosis (as evidenced by the transition from large macrovesicular to microvesicular droplets) and decreasing the overall MASH score. This suggests an intervention at an early stage of the disease, improving intrahepatic lipid metabolism and reducing hepatocyte stress. Second, at the biochemical level, the significant reductions in ALT and GOT levels in the Ob-CS and HE-Ob-CS groups indicate stabilization of the hepatocyte membrane and reduced cell death. This reduction in the release of liver enzymes into the bloodstream is a clear indication of the attenuation of the necroinflammatory process. The hepatoprotective mechanism of CS could be linked to the systemic metabolic effects observed in previous figures. The improvement in insulin sensitivity and the consequent low er flow of free fatty acids to the liver, together with the increase in adiponectin (a cytokine with hepatoprotective and anti-inflammatory effects), could be key factors contributing to the reduction in steatosis and liver inflammation.

## 3. Discussion

In this study, we demonstrated that administering a chickpea sprouts liquid diet to Wistar rats subjected to obesity induced by a hypercaloric diet and hypoestrogenism modified the distribution of adipose tissue, reduced the accumulation of mesenteric fat, and significantly improved the metabolic alterations associated with insulin resistance. These effects were accompanied by less progression to MASH.

The significant weight gain observed in the groups under a hypercaloric diet (Ob and HE-Ob) from the first weeks of the study is consistent with evidence that establishes an excess of carbohydrates promotes the expansion of adipose tissue through hypertrophy and hyperplasia. This phenomenon is driven by an increase in de novo lipogenesis at both adipose and hepatic levels. This mechanism is enhanced by the activation of key lipogenic transcription factors, such as SREBP-1c and ChREBP [23,24]. The activation of this transcriptional pathway facilitates triglyceride synthesis and subsequent accumulation in adipose tissue and peripheral organs, such as the liver, contributing substantially to body mass gain.

Notably, the HE-Ob group exhibited significantly accelerated weight gain during the first 8 weeks compared with the Ob group. This observation can be attributed to the loss of the protective metabolic action of estrogens. It is well documented that estrogens, acting predominantly through the ERα receptor, positively modulate the expression of genes involved in fatty acid β-oxidation and carbohydrate homeostasis by activating energy sensors such as AMPK and the transcription factor PPARα [25,26]. Therefore, a state of estrogenic deficiency, such as that induced in the HE models, predisposes to greater visceral fat accumulation, faster deterioration of insulin sensitivity, and reduced basal energy expenditure [27,28], thereby exacerbating diet-induced metabolic alterations.

The finding that the Ob group ultimately developed a higher final body weight than the HE-Ob group points to a complex temporal dynamic in the progression of obesity. While estrogen deficiency initially accelerates metabolic disruption and weight gain, ongoing energy overload in the Ob group, lacking any early compensatory mechanisms, eventually results in more severe obesity. This reinforces the notion that estrogens play a crucial role in initial metabolic resilience, and their absence unmasks and accelerates the pathophysiology of obesity; however, a sustained caloric load ultimately becomes the primary driver of adiposity in the long term [27,28].

The agreement between our findings and scientific literature reinforces the beneficial role of chickpeas and their derivatives in regulating carbohydrate metabolism. This effect has been classically attributed to its high content of soluble fiber, bioactive proteins, and phenolic compounds, which modulate intestinal glucose absorption and improve peripheral insulin sensitivity [22,29].

However, the marked effect observed with the intermediate dose of chickpea sprouts (CS; 0.9 g/kg) suggests a more specific mechanism of action, potentially mediated by its unique isoflavone profile, such as biochanin A and formononetin [30,31]. Germination significantly increases the concentration of these phytoestrogenic compounds [20,21]. Scientific evidence indicates that isoflavones improve insulin sensitivity by synergistically activating key signaling pathways, such as the IRS-1/PI3K/Akt pathway, and by modulating metabolic regulators, including PPARα and SIRT1 [16,17,21,28].

In addition to these agonist effects, CS exerts a crucial inhibitory action on metabolic inflammation. By reducing NF-κB activation, the synthesis of cytokines such as TNF-α and IL-6 is decreased [15]. This anti-inflammatory context is critical for restoring insulin signaling, as it prevents inhibitory phosphorylation in IRS-1 serine residues, a central mechanism in the pathogenesis of insulin resistance [32]. Thus, the CS acts dually, enhancing the anabolic pathways and suppressing the inflammatory signals that block them.

The specific reduction in caloric intake observed in the hypoestrogenic (HE-CS) group suggests a mechanism involving phytoestrogenic activity. The isoflavones in CS, such as biochanin A, may act as selective estrogen receptor modulators, centrally influencing appetite regulation. This selective effect could be mediated by the interaction of isoflavones, predominantly found in sprouts, such as biochanin A and formononetin, with estrogen receptors in the central nervous system, particularly in the hypothalamus [33]. Such an interaction could restore the balance between orexigenic (appetite-stimulating) and anorexigenic (appetite-suppressing) neuropeptides, whose regulation is altered in states of estrogenic deficiency [34].

Additionally, it is plausible that improved signaling of peripheral satiety hormones enhances this phenomenon. Evidence suggests that phytoestrogens and other bioactive components in chickpeas may affect hormone secretion and hormone sensitivity, such as leptin [29,35]. Therefore, the reduction in caloric intake in HE rats could result from dual actions: a direct central modulation of hypothalamic appetite circuits and a potentiation of peripheral satiety signals. These mechanisms synergize to restore energy homeostasis in the context of hypoestrogenism.

The increase in pancreatic weight observed in Ctrl and HE rats treated with nine-day-old chickpea sprouts (CS) may indicate compensatory β-cell hyperplasia. This phenomenon could be induced by the phytoestrogenic activity of the treatment, which has been reported to promote the proliferation and expansion of pancreatic islet mass by activating the alpha estrogen receptor (ERα) and cell survival and growth pathways, such as PI3K/Akt and MAPK [36,37]. The absence of this effect in obese groups (Ob-CS and HE-Ob-CS) suggests that an adverse metabolic environment, characterized by lipotoxicity and chronic inflammation, may exceed or limit the proliferative capacity induced by treatment, compromising the regenerative response of the pancreas [38]. However, it is plausible that the treatment exerts a protective effect, preserving the existing β cell mass from metabolic stress-induced apoptosis, thus contributing to maintaining a more adequate insulin secretion.

On the other hand, a significant reduction in lean mass was observed exclusively in the HE-Ob group in skeletal muscle. This finding suggests that estrogen deficiency alone is not enough to induce severe muscle atrophy, but it does dramatically increase the vulnerability of tissue to metabolic stresses such as obesity. Under normal conditions, the lack of estrogen partially impairs insulin sensitivity in the muscle by reducing the expression of GLUT4 and the activation of the PI3K/Akt pathway, without causing a significant structural loss [39,40]. However, when combined with a high-calorie diet, a lipotoxic environment is generated, characterized by intramuscular lipid accumulation and mitochondrial dysfunction, which amplifies insulin resistance and activates protein degradation mechanisms, ultimately leading to atrophy [26,41].

Insulin resistance in skeletal muscle is a fundamental defect in the pathophysiology of type 2 diabetes, as this tissue is responsible for approximately 75% of postprandial glucose uptake. An early deterioration in muscle glycogen synthesis is one of the first detectable metabolic alterations. It is considered the initiating defect and can precede β-cell failure and hyperglycemia by decades [42,43].

In this context, treatment with chickpea sprouts managed to maintain skeletal muscle mass in obese rats, even showing a tendency to increase. This protective effect could be attributed to an improvement in insulin sensitivity, which is achieved by enhancing the IRS-1/PI3K/Akt signaling pathway, a pathway essential for normal glucose uptake in muscle [44]. It is particularly interesting that the animals in the Ob group, despite having a higher lean mass compared to the HE-Ob group, exhibited the most severe metabolic alterations. This apparent paradox reinforces the notion that metabolic dysfunction and lipotoxicity can impact muscle mass, underscoring that the metabolic quality of tissue (its ability to respond to insulin and oxidize substrates) is a more critical determinant of carbohydrate homeostasis than muscle mass alone.

The significant expansion of the relative weight of adipose tissue in the Ob and HE-Ob groups confirms the development of obesity induced by the hypercaloric diet. The accumulation of subcutaneous fat is a characteristic phenotype of obesity resulting from energy excess, typically accompanied by adipocyte hypertrophy and the release of proinflammatory adipokines from visceral fatty deposits, such as the retroperitoneal and mesenteric regions [45].

Of particular interest is that the HE-Ob group had a greater accumulation of both visceral and subcutaneous fat compared to the Ob group, despite exhibiting lower body weight and reduced soleus muscle mass. This apparent discrepancy underscores the profound impact of estrogen deficiency on body fat distribution and muscle metabolism. The marked increase in visceral fat in this group, paradoxically, may indicate increased insulin sensitivity in this specific tissue, a finding consistent with previously reported HOMA index values. This paradox may be explained by the capacity of visceral adipose tissue to produce estradiol locally. This intracrine secretion could exert autocrine/paracrine protective effects, mitigating chronic inflammation and partially preserving insulin sensitivity within this depot [46,47,48].

Treatment with nine-day chickpea sprouts induced a noticeable change in fat distribution. In Ob rats, a redirection of lipid accumulation to retroperitoneal visceral deposition was observed, a change associated with improved insulin sensitivity at the systemic level. Previous studies have indicated that phytoestrogens can modulate the distribution of adipose tissue by activating estrogen and PPARγ receptors, thereby promoting adipogenesis in metabolically healthier deposits and reducing inflammation [49]. Additionally, these compounds exert systemic antioxidants and anti-inflammatory effects, including the activation of the Nrf2 factor and the inhibition of the NF-κB pathway, which results in a reduction in the levels of proinflammatory cytokines such as TNF-α and IL-6 [18,50].

In contrast, in the HE-Ob-Veh group, there was a greater accumulation of subcutaneous fat. This phenotype suggests the potentiation of a protective mechanism, possibly mediated by the synthesis of local estrogens in this tissue, which, by activating the ERα, PI3K/Akt, and MAPK pathways, improves insulin sensitivity and glycemic homeostasis [36,37,47]. Since the isoflavones in the sprouts share these mechanisms of action, it is plausible that no additional synergistic effect of the treatment will be observed in this specific parameter.

Finally, the treatment-induced reduction in the mesenteric fat of the Ob and HE-Ob groups is of great clinical relevance. This visceral deposition is closely linked to insulin resistance, systemic inflammation, and increased cardiometabolic risk [45]. Its decrease reinforces the beneficial role of chickpea sprouts in reconfiguring the body fat distribution profile towards a less pathogenic phenotype.

The results indicate that hypoestrogenism by itself promotes an alteration in glucose tolerance, without inducing systemic insulin resistance. This suggests that the initial damage is localized to specific tissues, mainly in skeletal muscle, as confirmed by the reduction in the weight of this organ in the HE-Ob group. This finding reinforces the notion that estrogen deficiency, while not sufficient to cause marked insulin resistance or muscle atrophy on its own, significantly increases tissue susceptibility to adverse metabolic stimuli, such as excess calories. It can be postulated that, under these conditions, it is a matter of time before generalized peripheral insulin resistance (in the liver and adipose tissue) develops. This progression correlates with the glucose tolerance curves (OGTT) of the HE and HE-Ob groups, where a higher glycemic peak was observed within the first 30 min and consistently elevated blood glucose concentrations throughout the entire test, compared to the Ctrl and Ob groups.

On the other hand, it is confirmed that a hypercaloric diet is a potent inducer of insulin resistance, as reflected in the increase in the HOMA index and the alterations in OGTT in the Ob and HE-Ob groups. This phenomenon is associated with compensatory hyperinsulinemia, in which insulin signaling through the IRS-1/PI3K/Akt pathway is impaired in muscle, liver, and adipose tissue, which favors lipid accumulation and peripheral lipotoxicity [51,52]. A particularly notable finding was the elevated concentration of fasting plasma triglycerides, which coexisted with fasting hyperinsulinemia and altered plasma triglyceride suppression. These data indicate the presence of marked adipocyte resistance to the antilipolytic effect of insulin in Ob animals [53].

The fact that the Ob and HE-Ob animals did not present frank hyperglycemia after five months of dieting, but did have hyperinsulinemia, indicates that they were in a prediabetic phase, where the secretory capacity of the pancreas was still able to compensate for peripheral resistance [54].

Notably, treatment with chickpea sprouts reversed these alterations, reducing insulin and triglyceride concentrations, and improving glucose tolerance in the Ob and HE-Ob animals, until reaching levels comparable to those of the Control group. This insulin-sensitizing effect may be mediated by the synergistic action of its bioactive compounds, such as isoflavones, saponins, and polyphenols. These compounds have been documented to activate key metabolic pathways such as AMPK, IRS-1/PI3K/Akt, PPARα, and SIRT1 [16,17,21,38,55]. Activation of these pathways would promote an increase in the expression of glucose transporters (GLUT4) in target tissues and a reduction in hepatic lipogenesis, thus restoring energy homeostasis.

The increase in adiponectin levels observed in the HE-Ob group treated with chickpea sprouts (CS) is consistent with an improvement in insulin sensitivity. This adipokine exerts its beneficial metabolic effects by activating the AMPK and p38 MAPK pathways, which promote fatty acid oxidation and reduce the inflammatory state in visceral adipose tissue [56,57]. The notable increase in subcutaneous adipose tissue in this group, in combination with treatment, could enhance this effect, as it has been described that the expansion of this fatty deposit plays a protective role by reducing chronic inflammation of visceral origin, thereby contributing to insulin sensitization at the peripheral level [58,59].

The dynamics of visceral adipose tissue are of relevance. The HE-Ob group developed obesity in a shorter period compared to the Ob group. This rapid expansion of visceral adipose tissue could induce a compensatory increase in the activity of aromatase, a key enzyme in the conversion of androgens to estrogens. The consequent increase in intra-adipose estrogen production could partially attenuate metabolic decline, reducing inflammation and modulating insulin sensitivity, thereby altering the secretory profiles of adipokines and inflammatory mediators. This mechanism is supported by reports highlighting the beneficial metabolic effects of increased adipose tissue aromatase activity [46,47,48]. It is plausible that these compensatory processes were activated during the intermediate stages of the obesity induction protocol (approximately halfway through the 10 weeks), which would explain the decrease in the rate of weight gain observed in the HE-Ob group after the first weeks of sucrose supplementation, contributing to a temporal modulation of metabolic deterioration.

In contrast, the Ob group presented the most severe metabolic imbalance. This phenomenon could be related to sustained hyperleptinemia, which inhibits GnRH secretion at the hypothalamic level, alters the pulsatility of LH and FSH, and impairs ovarian granulosa cell function, leading to a progressive decrease in estradiol synthesis [60,61]. This reproductive dysfunction secondary to obesity would explain the more unfavorable metabolic profile in the Ob group compared to HE-Ob and suggests that obesity itself constitutes a more severe determinant of long-term carbohydrate and lipid alterations than its combination with hypoestrogenism.

On the other hand, the CS-induced adipose tissue redistribution in the Ob group suggests that its hypoglycemic and insulin-sensitizing effect could be attributed to a greater extent to systemic antioxidant and anti-inflammatory mechanisms, such as the activation of the Nrf2 factor and the inhibition of the NF-κB pathway, with the consequent reduction in proinflammatory cytokines such as TNF-α and IL-6 [18,50] than direct phytoestrogenic action [62,63].

Taken together, these findings support a multifactorial action of chickpea sprouts, acting as an effective modulator of carbohydrate and lipid homeostasis in states of insulin resistance induced by obesity and hypoestrogenism.

Histomorphology results confirm that obesity and hypoestrogenism induce adipocyte hypertrophy, evidenced by an increase in cell size and a reduction in the number of adipocytes per field. This condition is directly associated with a proinflammatory state, characterized by adipose tissue dysfunction and infiltration of M1 macrophages. This microenvironment promotes the secretion of cytokines, such as TNF-α and IL-6, which impair insulin signaling by inhibiting the IRS-1/PI3K/Akt pathway, contributing substantially to the systemic insulin resistance observed in the Ob-Veh and HE-Ob-Veh groups [64,65].

Treatment with CS induced a remarkable phenotypic change in adipose tissue, increasing the number of adipocytes per field without significantly reducing the weight of visceral deposition. This finding suggests the promotion of the adipocyte hyperplasia process. The redistribution of lipids in a larger and smaller cell population is associated with better metabolic function, favoring adiponectin secretion, and reducing peripheral lipotoxicity [56,66]. This mechanism of “healthy” adipose tissue expansion is consistent with the concomitant improvement observed in glucose tolerance, insulin sensitivity, and triglyceride levels in the Ob-CS and HE-Ob-CS groups.

In parallel, a decrease in islet area was observed in the pancreas in the HE-Veh, Ob-Veh, and HE-Ob-Veh groups, confirming the combined deleterious impact of estrogen deficiency and insulin resistance on β-cell integrity. The chronic inflammation and lipotoxicity associated with these conditions compromise the viability and function of pancreatic endocrine cells [54,67]. The ability of the CS to significantly increase islet size in these groups suggests a proliferative and cytoprotective effect. This effect can be mediated by bioactive compounds in sprouts, such as isoflavones and polyphenols, which have been shown to activate cell survival pathways, including PI3K/Akt and ERK1/2, thereby counteracting cytokine-induced apoptosis and metabolic stress [68,69].

Overall, the dual action of the CS, promoting a hyperplastic expansion of adipose tissue and preserving the mass of the pancreatic islets, provides a solid mechanical basis to explain its efficacy in maintaining glycemic homeostasis and in delaying the progression to type 2 diabetes mellitus in models of obesity and hypoestrogenism.

Hepatic histopathological evaluation revealed a progression of damage from sinusoidal congestion and portal inflammation in the HE-Veh group, to macrovesicular steatosis and pericellular fibrosis in the Ob-Veh and HE-Ob-Veh groups, configuring a picture of steatohepatitis associated with metabolic dysfunction (MASH). This histological deterioration correlated with an increase in serum levels of alanine aminotransferase (ALT), a sensitive marker of necroinflammation and hepatocellular damage. These findings confirm that hypoestrogenism acts as a contributing factor that potentiates the lipotoxicity and chronic inflammation induced by the hypercaloric diet.

At the molecular level, this pathogenesis is characterized by dysregulation of liver metabolic programs. Activation of the lipogenic transcription factors SREBP-1c and ChREBP, which drive fatty acid synthesis, occurs concurrently with inhibition of PPARα, reducing β-oxidation capacity and leading to intracellular triglyceride accumulation [70,71].

Treatment with CS, enriched in isoflavones, saponins, and bioactive phenolic compounds, demonstrated a remarkable hepatoprotective effect. It reversed congestion and inflammation in the HE-CS group, promoted a transition of steatosis from macrovesicular to microvesicular in the Ob-CS and HE-Ob-CS groups, and significantly reduced serum triglyceride concentrations in both the HE-CS and Ob-CS groups. This improvement profile indicates a positive modulation of hepatic lipid metabolism, likely secondary to improvements in glucose tolerance and insulin sensitivity at both local (HE-CS) and peripheral (Ob, HE-Ob-CS) levels, which could redirect energy substrates toward muscle and liver glycogen synthesis [42].

The mechanism underlying this benefit could involve the activation of AMP-activated protein kinase (AMPK). AMPK activation inhibits lipogenesis, stimulates fatty acid β-oxidation, and attenuates the inflammatory response by suppressing the NF-κB pathway, which together contributes to attenuating steatosis and preventing the release of liver enzymes into the circulation [18,72].

Among the limitations of this study, the use of an animal model that does not comprehensively reproduce the physiology of the human female climacteric is identified. In addition, the intervention period with treatment (4 weeks) was relatively short to assess chronic effects, and the absence of more in-depth molecular analyses (such as quantification of receptor expression or transcriptomic studies) restricts detailed elucidation of mechanisms of action. Additionally, although the role of hyperleptinemia in ovarian dysfunction is discussed, the lack of direct measurement of leptin and 17β-estradiol concentrations limits the causal interpretation of this finding.

Future research should aim to validate these results in more complex preclinical models and clinical studies, and to determine the optimal dose, treatment duration, and potential synergistic interactions with other dietary components.

## 4. Materials and Methods

### 4.1. Plant Material

Kabuli-type chickpea seeds (*Cicer arietinum* L.) were procured from a commercial supermarket in Mexico City (Schettino brand). The seeds, sourced from Orizaba, Veracruz, Mexico, were light brown and angular, with an average weight of 0.52 ± 0.02 g and length of 0.4 ± 0.1 cm.

### 4.2. Germination Protocol

Chickpea seeds (250 g) were first imbibed for one hour to initiate germination. The seeds were then transferred to wide-mouthed glass containers, covered with cloth lids to facilitate gas exchange, and placed in an incubation chamber. The chamber was maintained at 28 ± 1 °C with a relative humidity of 45–65% and a 12 h light/dark photoperiod. Throughout the 9-day germination period, the atmospheric composition was rigorously controlled. Carbon dioxide (CO_2_) levels were maintained at 740–760 ppm via injection from a pressurized system, while oxygen (O_2_) was kept at 20.8 ± 0.5%. Both gases were continuously monitored using a gas analyzer (Thermo Scientific, Serie II Water Jacket (Thermo Fisher Scientific, Waltham, MA, USA) to prevent hypoxic stress and ensure optimal conditions. After germination, the sprouts were dehydrated at 45 °C for 24 h, pulverized, and stored at room temperature for subsequent analysis.

### 4.3. Preparation and Administration of Chickpea Sprouts (CS) Treatment

Germinated chickpea flour was suspended in water (30% *w*/*v*) using a magnetic stirrer, followed by one hour of sonication. This suspension (CS) was administered daily via intragastric gavage for four weeks at a dose of 0.9 g/kg/day. This dose was selected based on a prior acute treatment study, which demonstrated the most potent hypoglycemic effect compared to 0.3 and 3 g/kg. The vehicle control groups received an equivalent volume of water.

### 4.4. Animal Model and Experimental Design

Sixty-four female Wistar rats of 8 to 9 weeks of age, with an initial weight of 200 ± 10 g, clinically healthy and free of specific pathogens, were used and were obtained from the Department of Pharmacobiology of Cinvestav-IPN. The animals were housed under a controlled light/dark cycle of 12 h, with a temperature of 22 ± 2 °C and a humidity level below 60% with free access to water and standard diet. Animals showing signs of disease, wounds, loss of >20% body weight, non-treatment-induced metabolic disturbances, or accidental death were excluded. The removed animals were humanely slaughtered in accordance with the recommendations of NOM-062-ZOO-1999, and their data were excluded from statistical analysis. Rats were randomly assigned (systematically) to four primary experimental groups (*n* = 16 per group): Control (Ctrl), Hypoestrogenic (HE), Obese (Ob), and Hypoestrogenic-Obese (HE-Ob). Each primary group was subdivided into two subgroups (*n* = 8), which received either CS or vehicle treatment. Treatments were coded and administered by an independent technician; 669The researcher responsible for the analysis was unaware of the group assignment. The procedures and protocol No. 0087-14, approved on February 5, 2024, of this work were approved by the ethics committee (CICUAL-CINVESTAV-IPN), as well as the recommendations of the Official Mexican Standard (NOM-O62-ZOO-1999) for the specifications of techniques for the production, care, and use of laboratory animals [73].

### 4.5. Induction of Hypoestrogenism by Bilateral Ovariectomy

To create a hypoestrogenic state, the animals underwent a surgical removal of both ovaries at week 10. They were anesthetized using a ketamine-xylazine mixture (50–10 mg/kg, i.p.) (ANESTEK^®^ (AnesTek Corporation, Taoyuan City, Taiwan) and Procin^®^ (PiSA Agropecuaria S.A de C.V., Guadalajara, Mexico). The abdominal and pelvic areas were shaved and then disinfected with a 2% iodine solution. A longitudinal incision of approximately 1.5 cm was made to separate the skin from the muscle. Following this, a second incision of about 0.5 cm was made in the muscle to access the abdominal cavity. The fallopian tubes were identified, ligated near the uterus, and removed along with the ovaries. Both incisions were then sutured closed.

Postoperative care included an antibiotic (enrofloxacin, 10 mg/kg; i.m.) (Bayer, Leverkusen, Germany) and an analgesic (dipyrone, 200 mg/kg; i.m.) administered for three days. After one week of recovery, the animals were assigned to different experimental groups.

### 4.6. Induction of Obesity

Rats in the Obese (Ob) and Hypoestrogenic-Obese (HE-Ob) groups (*n* = 8) received a 30% (*w*/*v*) refined sucrose solution (Ingenio Azucarero Potrero, Veracruz, Mexico) as their drinking water *ad libitum* for 24 weeks, a period which included the 4-week SC treatment phase. Control (Ctrl) and Hypoestrogenic (HE) groups received filtered water *ad libitum*. All groups had free access to a standard solid diet (LabDiet 5008^®^). Daily measurements of food and liquid intake were recorded throughout the treatment period, with fresh supplies provided daily. This treatment began in week 12, which is considered the start of the experiment (week 0).

### 4.7. Evaluation of the Hypoglycemic Effect and Dose Selection

The sub-chronic dose (0.9 g/kg/day) was selected based on its superior hypoglycemic effect in an acute oral glucose tolerance test (OGTT) in control animals compared to 0.3 and 3 g/kg. For the OGTTs, rats were fasted for 12 h. A baseline blood glucose measurement was taken from the caudal tail vein using an Accu-Chek Active glucometer (Roche Diagnostics, Basel, Switzerland). Subsequently, the CS treatment (at the respective doses) or vehicle (water) was administered intragastrically. Ninety minutes later, an oral glucose load (2 g/kg) was administered, and blood glucose levels were monitored every 30 min for 120 min. An identical OGTT was performed at the end of the 4-week sub-chronic treatment to assess the sustained effect of CS in the different experimental groups.

### 4.8. Assessment of Insulin Resistance

Insulin resistance was estimated using the Homeostasis Model Assessment (HOMA) index, calculated with the formula: [fasting glucose (mmol/L) × fasting insulin (µU/mL)] / 22.5 [74]. Serum glucose concentrations were quantified using enzymatic-colorimetric diagnostic kits (Spinreact, Girona, Spain), with absorbance measured at 340 nm on a conventional spectrophotometer (Microlab 100, Merck KGaA, Darmstadt, Alemania). Serum insulin and adiponectin levels were measured using an enzyme-linked immunosorbent assay (ELISA) kit (MilliporeSigma, Burlington, MA, USA) according to the manufacturer’s instructions, with absorbance read at 450 nm on a spectrophotometer (Sunrise, TECAN, Tecan Austria GmbH, Grödig, Austria).

### 4.9. Biochemical Parameters

Serum concentrations of glucose, triglycerides (TGs), total cholesterol (CHOL), and the hepatic transaminases AST and ALT were determined using commercial diagnostic kits based on colorimetric principles (Spinreact^®^, Spinreact, SA., Bas, Girona, Spain). Spectrophotometric readings were performed on a Microlab 100^®^ instrument (Microlab 100, Merck KGaA, Darmstadt, Germany) at the wavelengths specified for each kit.

### 4.10. Morphological and Histological Evaluation

For histological analysis, tissue samples from the pancreas, liver, and retroperitoneal adipose tissue were fixed in 10% formalin-PBS (Sigma-Aldrich/MilliporeSigma, Burlington, MA, USA), dehydrated, and embedded in paraffin. The paraffin-embedded blocks were sectioned to a thickness of 4–6 µm using a microtome. Sections were stained with hematoxylin and eosin (H&E) and Sirius Red (Sigma-Aldrich/MilliporeSigma, Burlington, MA, USA) for microscopic examination. Images were captured using a Carl Zeiss light microscope (Carl Zeiss Model 63300, Carl Zeiss AG, Oberkochen, Germany) equipped with a Tucsen digital camera (9 megapixels) and TSview 7.1 software. Slides were observed at 10× and 40× magnification. Adipose tissue cell counts, as well as adipocyte and pancreatic islet diameter measurements, were performed by analyzing five randomly selected fields per sample.

### 4.11. Statistical Analysis

A sample size of 8 animals per group (8 groups, totaling 64) was established, with an expected difference of 20 mg/dL and a standard deviation of 15 mg/dL in glucose levels. The estimated statistical power to detect differences using ANOVA (α = 0.05) is approximately 0.6. This power is considered acceptable given the expected significant effect and exploration nature of the study. However, the aim was to minimize experimental variability by strictly standardizing conditions to maximize statistical sensitivity. Data are presented as mean (*n* = 8) ± standard error of the mean (SEM). All datasets were tested for normality using the Shapiro–Wilk test and for homogeneity of variances using Bartlett’s test. Statistical differences between groups were determined using one-way analysis of variance (ANOVA) followed by Tukey–Kramer’s post hoc test, as performed with GraphPad Prism version 6.0 (GraphPad Software, Inc, Boston, MA, USA). A *p*-value of less than 0.05 was considered statistically significant.

## 5. Conclusions

This research demonstrates that nine-day chickpea sprout supplementation exerts a multifaceted therapeutic effect in a rat model of obesity and hypoestrogenism, acting as an effective metabolic modulator. The treatment significantly reversed insulin resistance and hypertriglyceridemia, improved carbohydrate homeostasis, and promoted the redistribution of adipose tissue towards a more favorable phenotype by inducing adipocyte hyperplasia and increasing adiponectin levels. In parallel, it demonstrated a remarkable hepatoprotective effect by attenuating steatosis, inflammation, and hepatocellular damage, while also preserving and potentially expanding the pancreatic islet mass.

Based on observed metabolic improvements and the known bioactive profile of chickpea sprouts, we hypothesize that these benefits may be mediated by the synergistic action of their components, including isoflavones and polyphenols. While specific pathways such as AMPK/PI3K/Akt and NF-κB are plausible mediators, as discussed, our study did not directly measure these signaling cascades, and we acknowledge that other mechanisms may also be involved. Therefore, chickpea sprouts are postulated as a promising functional feeding strategy for the management of metabolic comorbidities associated with obesity and estrogen deficiency.

## Figures and Tables

**Table 1:** Energy consumption of experimental animals in the Control (Ctrl), hypoestrogenic (HE), obese (Ob), and obese hypoestrogenic (HE-Ob) groups, and those treated with chickpea sprouts (CS).

Parameters	Experimental Groups							
	Ctrl		HE		Ob		HE-Ob	
	Vehicle	CS	Vehicle	CS	Vehicle	CS	Vehicle	CS
Body weight (g)	295.4 ± 8.3	264.8 ± 6.2	361.4 ± 12.5	309.4 ± 8.3	609.7 ± 32.2 *^,a^	628.2 ± 38.1 *^,a^	568.8 ± 32.1 *^,a^	575.2 ± 47.9 *^,a^
Food Consumed ^1^	16.4 ± 1.1	12.4 ± 0.7	16.7 ± 1.4	10.9 ± 0.5 *^,a^	8.0 ± 0.8 *^,a^	7.0 ± 1.1 *^,a^	7.8 ± 0.7 *^,a^	7.2 ± 0.9 *^,a^
Fluid Consumed ^1^	37.0 ± 2.2	33.7 ± 2.7	36.8 ± 4.1	27.1 ± 2.7	46.5 ± 4.2	42.8 ± 4.0	49.1 ± 2.5	48.7 ± 5.3
Raw Intake (day)								
Total (Kcal)	52.4 ± 2.4	39.8 ± 1.2	47.9 ± 6.4	34.6 ± 1.0	80.9 ± 6.9 *^,a^	73.6 ± 5.3 *^,a^	83.6 ± 3.6 *^,a^	79.1 ± 5.8 *^,a^
Protein (g)	4.4 ± 0.2	3.3 ± 0.1 *	4.0 ± 0.5	2.9 ± 0.1 *^,a^	2.1 ± 0.2 *^,a^	1.9 ± 0.2 *^,a^	2.1 ± 0.1 *^,a^	2.1 ± 0.1 *^,a^
Protein (Kcal)	13.9 ± 0.6	10.6 ± 0.3 *	12.7 ± 1.7	9.2 ± 0.3 *^,a^	6.7 ± 0.5 *^,a^	5.9 ± 0.5 *^,a^	6.6 ± 0.4 *^,a^	5.9 ± 0.5 *^,a^
Fat (g)	2.7 ± 0.1	2.1 ± 0.1 *	2.5 ± 0.3	1.8 ± 0.1 *^,a^	1.3 ± 0.1 *^,a^	1.2 ± 0.1 *^,a^	1.3 ± 0.1 *^,a^	1.2 ± 0.1 *^,a^
Fat (Kcal)	8.6 ± 0.4	6.6 ± 0.2 *	7.9 ± 1.1	5.7 ± 0.2 *^,a^	4.2 ± 0.3 *^,a^	3.7 ± 0.3 *^,a^	4.1 ± 0.3 *^,a^	3.7 ± 0.3 *^,a^
Carbohydrates (g)	9.3 ± 0.4	7.1 ± 0.2	8.5 ± 1.1	6.2 ± 0.2	18.5 ± 1.6 *^,a^	16.8 ± 1.1 *^,a^	19.2 ± 0.8 *^,a^	17.6 ± 1.3 *^,a^
Carbohydrates ^2^	29.8 ± 1.4	22.6 ± 0.7	27.2 ± 3.6	19.7 ± 0.6	70.0 ± 6.1 *^,a^	63.9 ± 4.5 *^,a^	72.9 ± 2.9 *^,a^	67.0 ± 5.1 *^,a^
Energy intake (%)								
Protein	26.5	26.5	26.5	26.5	8.28 *^,a^	8.01 *^,a^	7.89 *^,a^	7.45 *^,a^
Lipids	16.5	16.5	16.5	16.5	5.19 *^,a^	5.02 *^,a^	4.90 *^,a^	4.67 *^,a^
Carbohydrates	56.8	56.8	56.8	56.8	86.52 *^,a^	86.46 *^,a^	87.20 *^,a^	84.70 *^,a^

Values represented as the average (*n* = 8) ± the S.E. One-way Anova. Post hoc Tukey. * = *p* < 0.05 vs. Ctrl-Veh. ^a^ = *p* < 0.05 vs. HE-Veh. ^1^ g/day, ^2^ Kcal.

**Table 2:** Relative organ weight (%) of experimental animals.

Parameters	Experimental Groups							
	Ctrl		HE		Ob		HE-Ob	
	Vehicle	CS	Vehicle	CS	Vehicle	CS	Vehicle	CS
Liver	2.50 ± 0.05	2.40 ± 0.09	1.90 ± 0.04 *	1.81 ± 0.04 *	2.20 ± 0.06	2.10 ± 0.08	2.01 ± 0.06 *	1.90 ± 0.06 *
Kidney	0.25 ± 0.01	0.29 ± 0.01	0.23 ± 0.01	0.22 ± 0.01	0.21 ± 0.01 *	0.20 ± 0.01 *	0.17 ± 0.01 *^,a^	0.18 ± 0.01 *^,a^
Heart	0.25 ± 0.01	0.27 ± 0.02	0.25 ± 0.01	0.27 ± 0.01	0.24 ± 0.01	0.26 ± 0.02	0.23 ± 0.01	0.25 ± 0.02
Pancreas	0.18 ± 0.02	0.32 ± 0.02 *	0.19 ± 0.02	0.31 ± 0.02 *^,a^	0.13 ± 0.02	0.13 ± 0.01	0.13 ± 0.01	0.12 ± 0.01
Soleus Muscle	0.05 ± 0.00	0.04 ± 0.00	0.05 ± 0.00	0.04 ± 0.01	0.04 ± 0.00	0.06 ± 0.01	0.02 ± 0.00 *^,ab^	0.03 ± 0.00 *
Subcutaneous fat	3.60 ± 0.60	1.60 ± 0.2	3.91 ± 0.51	4.82 ± 0.70	7.62 ± 1.10	8.61 ± 0.9 *^,a^	11.60 ± 1.10 *^,a^	11.11 ± 1.20 *^,a^
Perirenal Fat	2.90 ± 0.40	2.80 ± 0.4	4.01 ± 0.40	5.01 ± 0.31	6.41 ± 0.7 *	11.11 ± 0.51 *^,ab^	9.51 ± 0.51 *^,ab^	8.90 ± 0.71 *^,ab^
Mesenteric Fat	1.60 ± 0.20	0.72 ± 0.1	1.42 ± 0.11	1.13 ± 0.11	2.73 ± 0.4 *^,a^	2.40 ± 0.10	2.82 ± 0.20 *^,a^	2.22 ± 0.20
Total Fat	8.20 ± 1.20	5.21 ± 0.7	9.42 ± 1.12	10.90 ± 1.10	16.80 ± 2.0 *^,a^	22.22 ± 1.30 *^,a^	23.93 ± 1.50 *^,ab^	22.20 ± 2.00 *^,a^

The values represent the mean ± S.E. (*n* = 8) and are significantly different (*p* < 0.05) between the groups, as determined by the one-way ANOVA. Followed by Tukey’s test, * = *p* < 0.05 vs. Ctrl-Vehicle; ^a^ = *p* < 0.05 vs. HE Vehicle, and ^b^ = *p* < 0.05 vs. Ob Vehicle. Relative Weight (weight of the organ/weight of the animal × 100).

## Data Availability

The original contributions presented in this study are included in the article. Further inquiries can be directed to the corresponding authors.
